# Influence of Ingestion of Game Meat on Blood Concentration of Lead in Southern Germany: A Pilot Study

**DOI:** 10.1007/s00128-022-03661-w

**Published:** 2022-12-22

**Authors:** Martin Wepler, Jan Schreckenberg, Bastian Paul, Gebhard Fröba, Claus-Martin Muth

**Affiliations:** 1 Department of Anesthesiology and Intensive Care, Ulm University Medical Center, Albert-Einstein-Allee 23, 89081 Ulm, Germany; 2 Company Medical Service, Robert Bosch Automotive Steering Company, Schwäbisch Gmünd, Germany; 3 Department of Anesthesiology, Ernst von Bergmann Hospital, Potsdam, Germany

**Keywords:** Lead concentration, Blood, Game meat, Hunting

## Abstract

Consumption of game meat may exert additional lead exposure with potential health risks. The purpose of the present pilot study was to determine blood lead concentration in game meat and no game meat consumers in southern Germany. Concentration of lead in blood (µg·L^− 1^) was significantly higher in game meat consumers (n = 190; 21.3 [20.0; 29.7]) compared to study participants consuming no game meat (n = 74; 20.0 [20.0; 20.0], p < 0.0001). In study participants with no game meat consumption, blood lead concentration was significantly higher in those who perform active hunting (80.3 [50.5; 110.0]) as well as active shooting (80.3 [50.5; 110.0]) than in those with no hunting or shooting activities (20.0 [20.0; 20.0], p < 0.01). In conclusion, game meat consumers as well as active hunters and shooters should take in to account their potential for an increased lead exposure and the corresponding health risks.

## Introduction

For living beings as animals and humans, lead is the second most toxic metal after arsenic. Lead exerts its toxic effects via impairment of enzyme activities and cellular systems, especially during cell development, hematopoiesis, and reproduction (Assi MA 2016). Lead exposure to humans primarily takes place through ingestion as 20–70% of ingested lead is absorbed by the human body (Arias JA 2010). Some studies have reported a relationship between the activity of hunting (Tsuji LJS 2008b), the consumption of game meat per se (Iqbal S 2009, Morales JSS 2011), as well as the consumption of game meat shot with lead containing munition (Fachehoun RC 2015, Iqbal S 2009, Johansen P 2006) and blood lead levels of humans. The main purpose of the current pilot study was to investigate potential effects of game meat consumption on blood lead levels in a population in southern Germany.

## Methods and Materials

Between August 2013 and December 2014 study participants were asked to complete a questionnaire and agreed on taking one single venous blood sample (5-7ml). The adult (> 18 years) study participants were divided into two groups (game meat consumption and no game meat consumption). In both groups, blood lead concentration was measured via a single venous blood sample. For analysis of blood lead level, a special cannula was used (S-Monovette® Safety-Canula, ØxL: 21G x 1 1/2’’, 38 mm long, sterile, free of pyrogen and endotoxin, Sarstedt, Nümbrecht, Germany) to not contaminate blood samples with additional lead. The participant’s blood sample were analyzed at the department for clinical chemistry at the University Hospital Ulm, Ulm, Germany. The lithium-heparin full blood was diluted 1:10 with blank value solution for lead, vortexed and left for at least one hour for hemolysis. After vortexing again, blood samples were ready for analysis at room temperature for seven days (Schmitt Y 1987). Concentration of lead was measured via graphite furnace atom absorption spectroscopy. The lowest detection threshold was 20 µg·L^-1^. Therefore, it was not possible to detect lower levels than 20 µg·L^-1^ so that this value also corresponds to zero lead concentration in blood. If concentration of lead was > 200 µg·l^− 1^, the sample was diluted to a concentration within the calibration range and the result was calculated under consideration of the dilution factor. Data sets were analyzed using non-parametric statistics. P-values < 0.05 were considered statistically significant. Quantitative graphical presentations and statistical analyses were accomplished by using GraphPad Prism 8 (GraphPad Software Inc, La Jolla, USA).

## Results

**Fig. 1:**
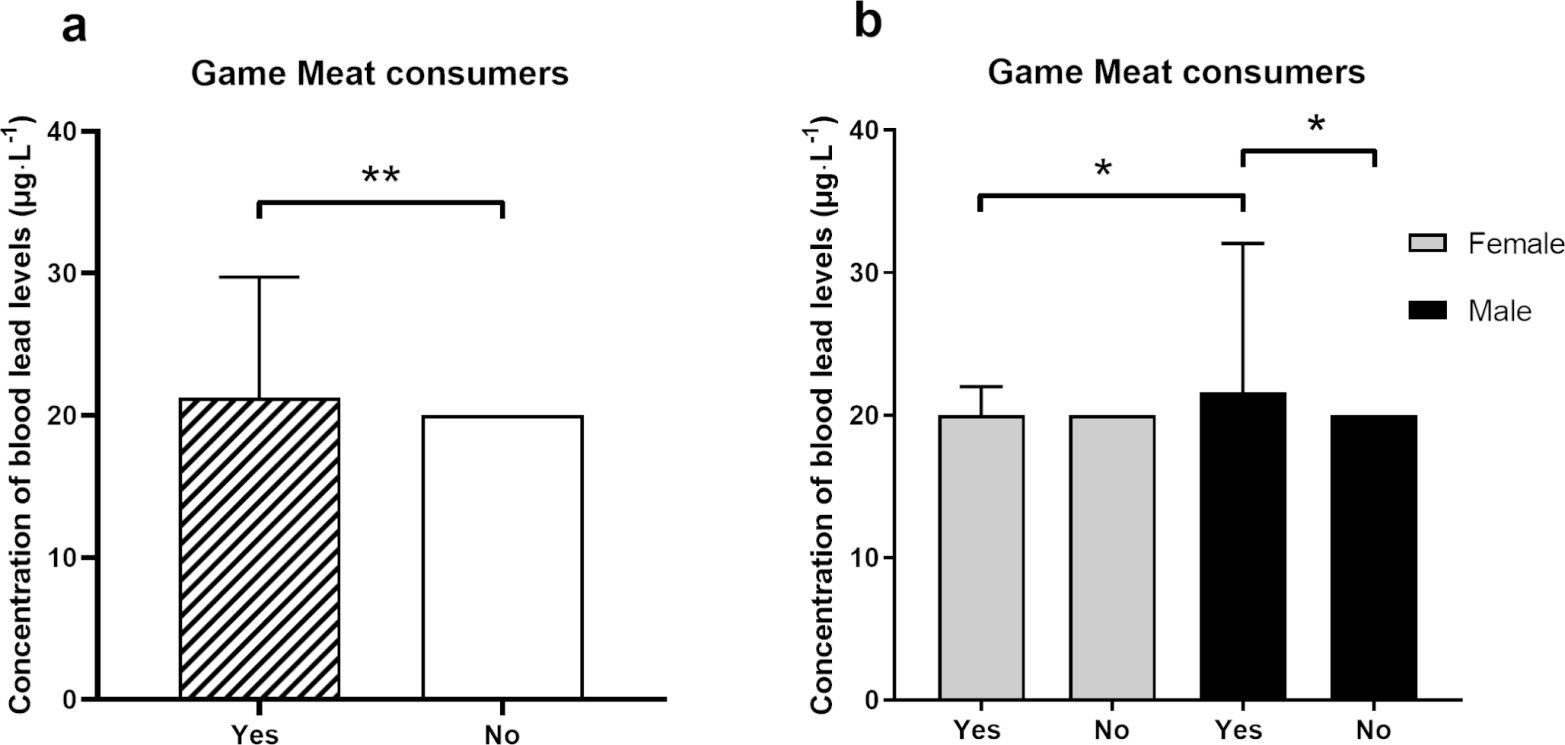
Concentration of blood lead levels and consummation of game meat. **(a)** Concentration of blood lead levels in all study participants divided by game meat consumers and no game meat consumers. Data is presented as median and interquartile range (25^th^ and 75^th^ percentile). **=p < 0.0001 for unpaired Mann-Whitney-Test. **(b)** Concentration of blood lead levels in game meat consumers and no game meat consumers separated by gender. Data is presented as median and interquartile range (25^th^ and 75^th^ percentile). *=p < 0.05 for Kruskal-Wallis-Test with Dunn’s post hoc test.

**Fig. 2:**
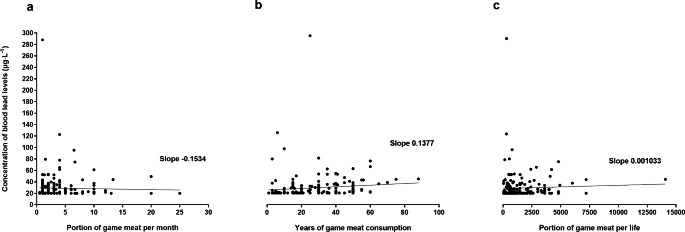
Concentration of blood lead levels in game meat consumers in relation to the amount of game meat consumption. **(a)** Concentration of blood levels and consume of game meat portions (150 g per portion) per month as well as **(b)** years of game meat consumption, and **(c)** per life in game meat consumers. Portions of game meat per life are determined via consumption of game meat portions per year multiplicated with years of game meat consumption. Each dot represents blood lead concentrations for one study participant (game meat consumers only). Lines represent simple linear regression analysis with slope presented.

**Fig. 3:**
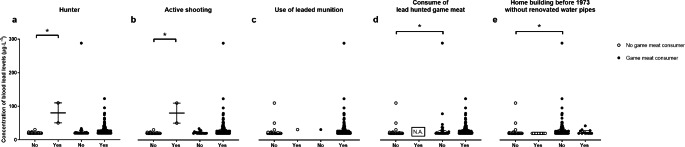
Shooting activities and other lead expositions in game meat and no game meat consumers. **(a)** Concentration of blood lead levels for game meat consumers and no game meat consumers who are hunters, **(b)** perform active shootings, **(c)** use leaded ammunition, **(d)** consume lead hunted game meat, and **(e)** live in houses build before 1973 without renovated water pipes. The year 1973 was chosen because since that timepoint it was not permitted any more to build new houses with lead-containing pipelines in Germany. Each dot represents blood lead concentrations in one study participant. *p < 0.01 for Kruskal-Wallis-Test with Dunn’s post hoc test.

After informed consent, a total of 265 study participants could be included in the study. Due to a measurement problem regarding blood lead levels and therefore one missing blood lead concentration, one study participant was excluded from the study. Of the other 264 study participants, all send back questionnaires, which also all could be analyzed. Baseline characteristics as well as number of hunters, number of active shooters including the use of leaded ammunition and living conditions are presented in Table 1. Concentration of lead in blood was significantly higher in game meat consumers compared to study participants consuming no game meat (Fig. 1a). Concentration of lead in blood was higher in male game meat consumers then in male study participants who consume no game meat (Fig. 1b). Additionally, blood lead concentration was higher in male game meat consumers then in female game meat consumers (Fig. 1b). Figure 2 shows blood lead concentrations in all game meat consumers in relation to portions of game meat over time. Concentrations of blood lead levels for game meat consumers and no game meat consumers with different living activities are presented in Fig. 3.

**Table 1:** Study population. Values are presented as median and 25^th^ and 75^th^ quartile or percent as stated.

	Game meat consumers	
	Yes (n = 190)	No (n = 74)
Sex (female)	26 (17%)	42 (57%)
Age (years)	54 (46; 66)	43 (30; 58)
Active hunter (n)	164 (86%)	2 (3%)
Active shootings per month (n)	4 (2; 6)	0 (0; 0)
Use of leaded ammunition (n)	183 (96%)	0 (0%)
Residential home build before 1973 (n) - with renovated pipelines (n)	26 (17%) 31 (16%)	17 (23%) 8 (11%)

## Discussion

The present study was performed as a pilot study and is, to our knowledge, the first evaluating blood lead level in game meat and no game meat consumers in southern Germany. The results of this pilot study indicate that in a small population in southern Germany the consumption of game meat may increase blood lead concentration (Fig. 1a), which was more likely in male game meat consumers then in female game meat consumers. With regards to the gender, it must be considered that only 17% of the game meat consumers in the present study were female, whereas 57% of the no game meat consumers were female (Table 1). The consumption of game meat has also been described to increase lead exposure in a human population in Spain (Morales JSS 2011). However, in the present pilot study, blood lead concentrations did not seem to correlate with the amount of game meat consumed per month or per life, but only for years of game meat consumption (the latter with a slope > 0 in the linear regression, Fig. 2), which may indicate that a more permanent consumption of game meat may exert an increasing effect on blood lead level instead of a more occasional consumption. With regards to blood lead level and game meat consumption it must be considered that the use of lead containing ammunition for hunting may further increase lead concentration in game meat. Although the United States and several European Countries have banned lead containing ammunition for hunting waterfowl for over twenty years now (Tsuji LJS 2008a), lead containing ammunition is still widely used for hunting big game and small game animals, which may increase lead concentration in their meat (Ertl K 2016) and therefore in the consumers of such meat. It must be noted that in the current literature there are high contradictoriness with regards to lead contamination (human and wildlife) from ammunition. In the present pilot study, we cannot exclude that other factors, e.g. environmental factors, may have influenced lead concentration of game meat in our study population. However, in the present pilot study consumption of lead hunted game meat did not show a significant effect on blood lead levels within the group of game meat consumers (Fig. 3). In contrast, active hunting or shooting showed an effect on blood lead levels within the group of no game meat consumers, but these results must be interpreted with caution, because there were only two study participants in the no game meat consumption group with shooting or hunting activities (Fig. 3). The living status with regards to lead containing pipelines did not seem to have influenced blood lead level in our study population (Fig. 3). Overall, when analyzing the blood lead concentrations in the present pilot study, the significantly higher blood lead level within the game meat consumers compared to no game consumers have also to be interpreted with caution. Although the difference between game meat and no game meat consumers was statistically significant, the difference per se is very low and therefore might biologically not be relevant. In Germany, the German Research Foundation (Deutsche Forschungsgemeinschaft, DFG) regularly publishes a list with reference values for the maximum allowable concentration at the workplace as well as for the biological reference value for workplace substances including lead. For 2020, the biological reference value for lead was 30 µg·L^− 1^ for women and 40 µg·L^− 1^ for men (https://mak-dfg.publisso.de/). In the present pilot study, only two female study participants (one game meat and one no game meat consumer) had blood lead concentrations above 30 µg·L^− 1^ (0.76%). Within the male study participants, 25 had blood lead concentrations above 40 µg·L^− 1^ (9.5%). However, only 2 of these male study participants were no game meat consumers and 23 were game meat consumers. Furthermore, for blood lead concentrations above the biological tolerance value, the DFG lists a biological reference limit, which describes a threshold for which certain protection manners must be used at the working place. In the 2020 list, this value was 200 µg·L^− 1^ for lead blood concentration (https://mak-dfg.publisso.de). In the present pilot study, only one male study participant showed a blood lead concentration above this limit (288 µg·L^− 1^). Although he was a game meat consumer, he also was an active sports marksman at the IPSC (International Practical Shooting Confederation) with many practical training units at short shooting distances and the use of lead containing ammunition, which was most likely the main reason for such a high blood lead concentration in this individual.

A limitation of the present pilot study may be that hobbies other than hunting and shooting sports, e.g. stained glass or ceramics, or residential and workplace proximity to potential sources of lead were not included. Also smoking as a potential contributor to blood lead levels (Richter PA 2013) was not assessed in the present pilot study and should be incorporated in future studies addressing effects on blood lead level in humans.

In conclusion, concentration of lead in blood was significantly higher in game meat consumers compared to study participants consuming no game meat. Additionally, in study participants with no game meat consumption, blood lead concentration was significantly higher in those who perform active hunting as well as shooting. However, overall differences in lead blood concentrations were very low and mostly below the biological tolerance value of 200 µg·L^− 1^. Nevertheless, game meat consumers as well as active hunters and shooters should take in to account their potential for an increased lead exposure and the corresponding potential health risks. Monitoring of blood lead level may detect concentrations above the recommended threshold.
